# Lord Cromer and Improved Hygiene in Egypt

**Published:** 1907-06-29

**Authors:** 


### SPECIAL ARTICLE.

### LORD CROMER AND IMPROVED HYGIENE IN EGYPT.

Now that Lord Cromer has relinquished the reins of administration abroad, and has been welcomed by his King and countrymen with warm appreciation upon his permanent return home, it may be interesting to recall the medical and sanitary developments which have taken place in Egypt since he went there in the autumn of 1883 as Sir Evelyn Baring. He found the country paralysed from the crushing effects of civil war, bankruptcy, native incompetence, cattle plague, and a devastating epidemic of cholera, which latter had thoroughly proved the inefficiency of the native public health officials. A new Public Health Department was created by Mr. Clifford Lloyd, an Englishman was placed at its head, and it has remained under British guidance ever since, while both the British and European staff have been gradually increased as occasion required. When cholera again visited Egypt it found officials more or less prepared, various subsidiary administrations existing to check its advance, laws to deal with infected localities, temporary hospitals for isolating the sick and, most important of all, a water supply in the chief towns which could be guarded from contamination.

### Fighting Plague.

It is now exactly eight years since plague reached Alexandria for the first time since Kinglake wrote his "Eothen," and though the epidemics have temporarily died down only to reappear every year in many districts, the disease has been well controlled by the energy of British health officers, and has never again spread in the way it did in classical times in Egypt, or as, unfortunately, it has been allowed to blaze in India during the present pandemic. The success which has attended plague measures in Egypt is somewhat emblematic of Lord Cromer's own success in general administration. The rules adopted have been never to despise the enemy, never to neglect an opportunity of attacking the individual unit, and always to carry out measures as thoroughly as possible. We may here mention that it is probably the universal destruction of rat parasites, at a time when they were not recognised as the chief vehicles by which bubonic and septicæmic plague are conveyed, that Egypt owes her comparative freedom from this terrible disease.

### The Campaign against Cholera.

Cholera and many other diseases have been imported during recent years by pilgrims returning from Mecca, often in the summer season when infectious diseases are extra prevalent, and it therefore became necessary to issue regulations for the control of the Hadj in so far as Egyptian Mussulmans are concerned. All pilgrims are now carefully examined, washed, and their property disinfected, while supervision of steamers insures their travelling in comparative comfort to and from Jeddah, though they are still plundered by Beduin and poisoned by sewage-infected water on Turkish territory. The quarantine service, which has also been under British chiefs during Lord Cromer's rule, has made considerable strides towards the effective exclusion of exotic disease, both human and epizootic, and much more could have been done if the service had not been a relic of internationalism, governed too often by political instead of scientific considerations.

### The Institute for Hygiene.

An Institute for Hygiene was erected in the garden of the Public Health offices, presided over by disciples of Professor Koch, who, it may be remembered, discovered the cholera bacillus in Egypt and has paid that country many visits since. In connection with this Institute doubtful cases of diphtheria are examined free of charge, and disinfection of houses is also carried out gratuitously; many cases of enteric and Mediterranean fever have been diagnosed by the serum test, and cerebro-spinal fever and other diseases have also been examined. Within a few yards stands a well-appointed Vaccine Institute, where buffalo calves supply sufficient material to safeguard Egypt and the Sudan from small-pox. Within the last year another separate Institute for counteracting rabies has been opened by the Government, in succession to one which was formerly managed by an Italian society; for hydrophobia is still well known in Egypt. A special hospital for infectious diseases exists permanently in the desert near Cairo, while in every smaller town there are temporary huts for that purpose, which can be easily burnt when done with. Space does not allow us to refer to the gigantic improvement of the Irrigation Department, on which a supply of pure drinking water partly depends, but we may mention that Cairo is now supplied with several deep, protected wells, which give better water than that formerly taken from the Nile. Alexandria has a successful installation of Jewell filters and some of the smaller towns are already well provided. Cattle plague has been almost stamped out, thanks to serum concocted in the desert, and a veterinary school has recently been established.

### Egyptian Hospital Reform.

But perhaps the greatest reforms accomplished have been in the various government hospitals; from a disgrace, every one has now become a credit to the district which owns it, while Kasr el Ainy, with its British medical and nursing staff, has the reputation of being one of the best appointed Oriental hospitals of modern times. The various ophthalmic hospitals are fighting dirt and prejudice, and the lunatic asylum, where the unfortunate inmates used to be loaded with chains until they died of tuberculosis or dysentery, has been so enlarged and reformed that it is becoming difficult to trace the spots where the horrors of twenty years ago were enacted. The School of Medicine has passed through many vicissitudes since it was opened under French influence some seventy years ago. To-day the students are taught in the English language by well paid professors, whose posts are eagerly competed for as vacancies arise. The school curriculum has been recognised by the Conjoint Board of London, who send an assessor to attend the annual examinations. It is not too much to say that the teachers of anatomy and helminthology have made discoveries during the last few years which are of importance to the whole world. We might write at additional length on the great improvement which has taken place in the physique of medical and all other students, the reformed prisons, whence relapsing fever has been banished by destroying the countless bugs which used to inhabit them, the establishment of the anthropometric system in the police offices, the cheapening of salt, which it is hoped may lessen the great scourge of pellagra, the various improvements which have taken place in fighting malaria in the Sudan and the establishment of the Wellcome research laboratory at Khartoum, which has already produced two splendid reports.

Although the future historian, when describing the success of Lord Cromer's policy and administration in Egypt and the Sudan, will not be tempted to place the measures which we have here sketched in the foreground of his greatest achievements, it must always be remembered that the technical workers, who have been able to carry sanitary reform to its present point, must have lamentably failed if they had not been constantly supported by his advice and encouragement.

